# Wet collections accession: a workflow based on a large stonefly (Insecta, Plecoptera) donation

**DOI:** 10.3897/BDJ.6.e30256

**Published:** 2018-12-07

**Authors:** R. Edward DeWalt, Matthew Yoder, Elise A. Snyder, Dmitry Dmitriev, Geoffrey Donald Ower

**Affiliations:** 1 Illinois Natural History Survey, Champaign, United States of America Illinois Natural History Survey Champaign United States of America; 2 University of Illinois, Champaign, United States of America University of Illinois Champaign United States of America

**Keywords:** wet collections, donations, imaging, digitization, natural history specimens

## Abstract

This study details a workflow used to accession a large stonefly (Plecoptera) collection resulting from several donations. The eastern North American material of Kenneth W. Stewart (deceased, University of North Texas), the entire collection of Stanley W. Szczytko (deceased, University of Wisconsin, Stevens Point), and a small portion of the Barry C. Poulton collection (active, United States Geological Survey, Columbia, Missouri) were donated to the Illinois Natural History Survey in 2013. These 5,767 vials of specimens were processed to help preserve the specimen legacy of these world renowned Plecoptera researchers. The workflow used an industrialized approach to organize the specimens taxonomically, image the specimens and labels, and place the specimens into new storage. Utilizing the images as a verbatim data source, we transcribed labels in iterative steps that yielded more information with each pass. The data were normalized, locations georeferenced, all specimen data formatted to meet Darwin Core Archive format for occurrence data, and a data set created using Pensoft's Integrated Publishing Toolkit. This is the first time that any of the specimen data has been made available electronically. We also provide two important electronic supplements that include the Bill P. Stark (active, Mississippi College) Oklahoma field notebook for 1971 and 1972 detailing locations for many coded stonefly specimens in the Stewart collection, and the coded locations of B. C. Poulton's Arkansas and Missouri study. Again, we have linked coded labels in vials to normalized and georefenced site data. We confirmed 243 stonefly species were contained within the collections, and the potential for many more species exists among the specimens identified to family and genus level. Twenty-one new state, province, and other significant stonefly records are reported herein with all identifications verified by the senior author, often through consultation with other stonefly taxonomists. Researchers are encouraged to utilize the specimen data, form collaborations with the authors, and borrow specimens for research.

## Introduction

Entomological research collections are often asked to accept donated material from other institutions and individuals. Recent times have seen smaller collections being closed due to institutional change in emphasis. Acceptance of material from private and small collections into larger institutions has benefits since these sources often improve geographic coverage and taxon representation of receiving institutions (Casas-Marce et al. 2012). Accepting worthy donations also protects against the loss of the lifetime work of amateur and professional taxonomists. However, donations impose burdens on accepting institutions in the form of additional space needs, staff time allocated, and the need for additional funding. The material often requires quarantine and fumigation for pest control, sorting and identification, improvement in labeling, and transfer into storage systems particular to receiving institutions. To improve the usefulness and availability of the specimens, digitization of the label data may also be necessary. A revolution in digitization of museum specimens is well underway (Page et al. 2015) and recognition of common tasks and workflows has been outlined that will improve the efficiency of specimen digitization (Nelson et al. 2012).

Current workflows for digitizing pinned arthropod material are maturing to utilize automated technologies to capture images (Dietrich et al. 2012, Hudson et al. 2015) and often involve transcription of labels through crowd sourcing (Blagoderov et al. 2012). Comparatively, digitization of wet insect collections is more difficult and the workflows much less automated, which presumably has led to fewer institutions digitizing wet collections. Fortunately, iDigBio has led workshops (iDigBio 2013) and provided guidelines for wet specimen digitization (iDigBio 2014), though few published workflows are available, and to our knowledge, none have been published specific to wet insect collections.

Donations of wet specimens often arrive in a large variety of storage types including WhirlPacTM bags , baby food jars, and museum jars of varying sizes. Sorted specimens may arrive in nested containers with cotton-stoppered shell vials inside, glass patent lip vials, and screw cap vials of wide variety and volume. Closures for vials also vary greatly and include rotting or melting stoppers, paper lined screw caps, and even cork stoppers. Preservatives include formalin based fixatives, alcohols of varied types and concentration, and even embalming fluid. We often do not know what idiosyncratic preservation techniques have been used to affect specific tissue conditions. Donated specimens may be in great condition or in a state of decay due to fluid evaporation from failed closures. Others may be coated in latex residue resulting from melting stoppers. It becomes a major challenge to weed out damaged specimens and move those worthy of accession to appropriate storage that is both unifying for the host institution and capable of protecting specimens in perpetuity.

Since 2013 the Illinois Natural History Survey (INHS) has accepted donations in excess of 56,000 wet arthropod specimen units from several private collections and two small public institutions (Ohio Biological Survey and Southern Illinois University Carbondale). The United States National Science Foundation (NSF), Division of Biological Infrastructure (DBI), Collections in Support of Biological Research (CSBR) Program (NSF DBI 14-58285) has provided funding to accession the specimens, capture images of specimens and labels, and transcribe the label data of this material into the INHS Insect Collection. In this paper we present the workflow used to accession nearly 6,000 vials of stoneflies (Insecta: Plecoptera), an order of aquatic insects highly sensitive to water and habitat quality changes (Lenat 1993). Nearly 4,000 extant, valid species are recognized globally, while in North America (Canada, Mexico, and the United States) approximately 780 species and subspecies are recognized (DeWalt et al. 2018b). The stonefly donations included all the eastern North American Plecoptera specimens of Kenneth W. Stewart (deceased), all the Plecoptera specimens of Stanley W. Szczytko (also deceased), and a small subset of the Barry C. Poulton (active) collection.

The objectives of this paper are to:

1. Document the workflow used for efficiently accessioning and digitizing these wet collections.
2. Provide access to two key digital documents that allow association of event data with a large proportion of the donated specimens.
3. Characterize the taxa present, the geographic extent of the donated specimens, and any significant records within the donated Plecoptera material.
4. Discuss strengths and deficiencies of the specimen data, including how the specimens and data might be used in the future.
5. Provide access to all specimen data in Darwin Core Archive (DwC-A) format.

### Importance of donors

**Kenneth W. Stewart** of the University of North Texas, Denton, died 9 December 2012. Stewart worked on a wide range of taxonomic, behavioral, and ecological aspects of stonefly biology. Ken's passing was mourned by students of aquatic insects the world over. Readers should consult Stark and Baumann (2013) for an obituary and list of publications. The US National Science Foundation (NSF) supported his efforts with three grants (DEB 78-12565, BSR 83-08422, BSR 85-055881) that led to the writing of his nymphal keys (Stewart and Stark 1988, Stewart and Stark 2002), a monograph of the stoneflies of the Ozark and Ouachita mountains (Poulton and Stewart 1991), and many other smaller papers. Many reared specimens and important geographic specimen records resulted from Stewart's 50 year career, which we secure for future use. Stewart offered his collection to both Brigham Young University (BYU) and the INHS, institutions with exceptionally large and historically important collections of stoneflies. The eastern specimens (3,782 vials), those collected from the Colorado/Kansas border (approximately -102 longitude) eastward, went to the INHS, the remainder going to BYU. Some crossover in destinations was inevitable. Several hundred vials of Stewart specimens were provided with only coded event (date/location information) data. Luckily, ledgers with detailed documentation were available. A field notebook with event data related to Oklahoma studies (Stark and Stewart 1973a, Stark and Stewart 1973b) and a key to Arkansas and Missouri (Poulton and Stewart 1991) locations were digitized and linked to specimen records.

**Stanley W. Szczytko** retired from the University of Wisconsin Stevens Point in May 2013. That spring he donated 1,736 vials of stoneflies to the INHS. His collection emphasized the mega-diverse genus *Isoperla* (Szczytko and Stewart 1979, Szczytko and Kondratieff 2015) and related genera. Also included were 547 vials of stoneflies from Wisconsin. Szczytko was a co-PI on DEB 85-05881 during 1985‑1988, in which vibrational communication (drumming) was investigated as a behavioral isolating mechanism for stoneflies, a project that generated several hundred reared stonefly specimens and associated drumming signals. Due to his long-term revision of the Nearctic *Isoperla (Szczytko and Stewart 1979, Szczytko and Kondratieff 2015)*, Szczytko held in his collection some of the most important INHS *Isoperla* primary types and many other specimens, all loaned prior to our specimen digitization efforts. These specimens have now been repatriated and their data made available through this paper. Stan died in a boating accident in September, 2017. DeWalt and Kondratieff (2018) provided an obituary and a list of Szczytko's publications.

**Barry C. Poulton** works for United States Geological Survey in Columbia, Missouri. He maintains a large collection, but a small portion of it (249 vials) was donated to the INHS in 2013. His most important stonefly work was a faunistic survey and watershed based analysis of stoneflies of the Ozark and Ouachita Mountains that yielded thousands of vials of material (Poulton and Stewart 1991). Many of these specimens were left in the care of Stewart. Some have been distributed to several stonefly researchers, while others remain in Poulton's collection in Columbia. Some are thought to be in the possession of the Elm Fork Museum of Natural History, Denton, Texas, but repeated attempts to document this have failed. Digitization of Poulton's Arkansas and Missouri sampling locations will allow those who have his specimens to link them with locations for the first time.

## Workflow and Methods

**Initial condition.** Initial profile scores (Favret et al. 2007) for this material indicated that identification level, processing status, and potential data quality scored in the top 75th percentile. Conversely, arrangement level, label condition, conservation status, and several other metrics scored at 50% or less. Key detractors for condition were low and dark ethanol levels, poor stopper condition, an unknown mixture of preservatives, labels with coded collection events, and a wide range of storage types and sizes that would prevent easy accession.

**A little planning prevents false starts.** At the beginning of the grant we spent two months discussing and testing workflows. It was our aim to accession these specimens and improve the profile scores dramatically. Most important was the replacement of preservatives and vials that were in poor condition. In addition, we noted that many labels were failing, e.g., letters were susceptible to flaking or rubbing off if handled carelessly. We did not believe we had the time to replace labels, but we could associate an indelible catalog number with each vial that would provide a permanent link to the specimen data. Imaging of specimens and existing labels would provide the verbatim data source, allowing reinterpretation of labels at any time.

Imaging adds time to digitization but provides a superior product. How do we save time while imaging? A standard photographic jig could be used to regionalize specimens and labels of various types, minimizing time spent searching for particular types of labels (e.g., catalog, determination, event, and others labels). How do we accomplish all the curatorial work and obtain useful images for so many vials? After testing (1) a single vial at a time, (2) a round robin approach where three vials were worked on simultaneously, and (3) an industrialized approach where multiple racks of vials were focal units, it was readily apparent that we gained efficiency of scale by simultaneously processing large batches of vials, up to 10-12 racks at a time We settled on a workflow that industrialized the process, minimized handling and damage of specimens, and accomplished all the curatorial tasks to increase longevity of specimens. The workflow was done in stages (pre-imaging, imaging, post-imaging, and transcription) that could be conducted by undergraduate student labor and stopped after the completion of a particular phase without risking damage to the specimens.

**Figure 1.**
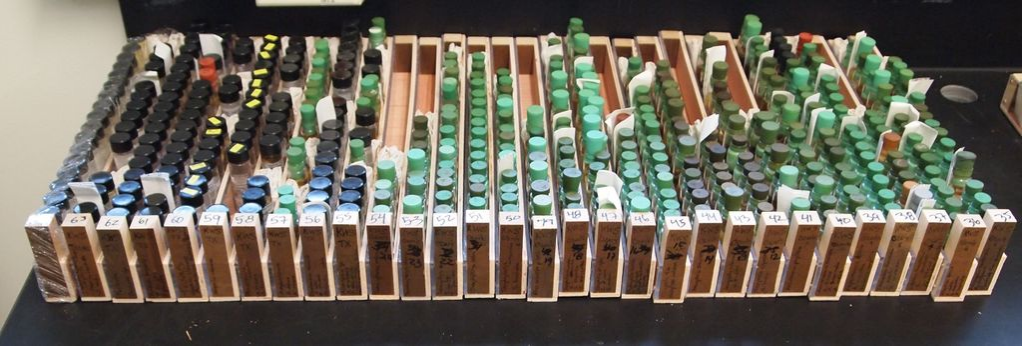
Plecoptera specimens in original vials sorted by taxon into consecutively numbered vial racks.

**Pre-imaging.** Specimens were sorted by taxon and placed into consecutively numbered, custom built, 43 cm X 2.8 cm wooden and Plexiglas_TM_ vials racks (Fig. 1). These vial racks were used as units for all subsequent curatorial processes, although separation by donor group was maintained to simplify metadata generation. At the end of these processes all specimens and labels would reside in 3 or 4 dram, glass screwcap vials with caps fitted with beveled plastic liners, therefore unifying storage for the donated wet specimens.

**Figure 2.**
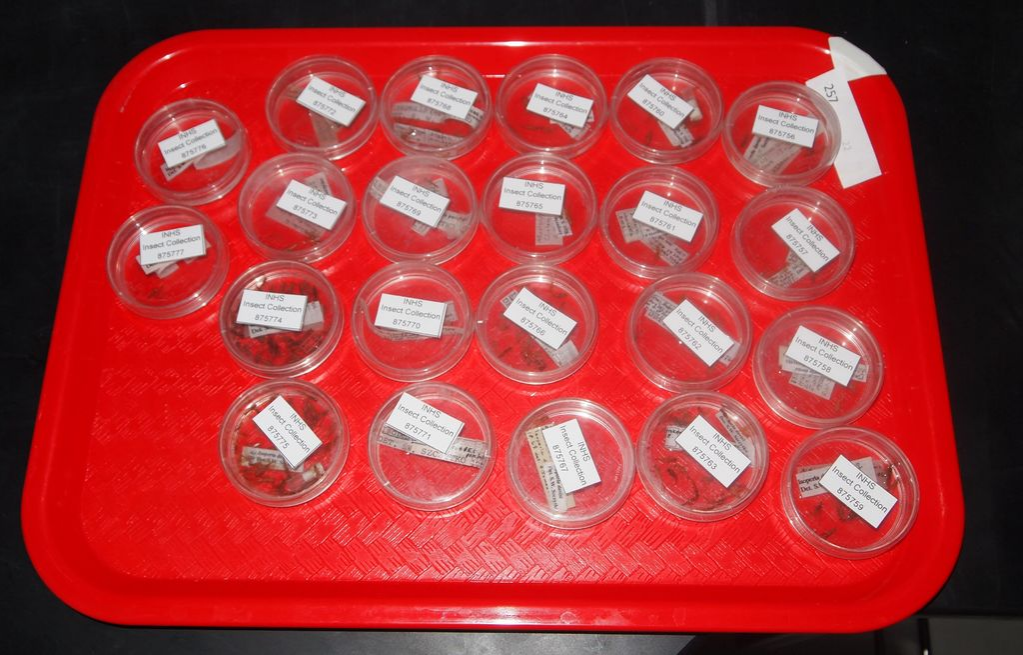
Tray and Plecoptera specimens ready for imaging. Note metadata label in upper right corner detailing rack number and number of vials. White tape in that corner orients placement of dishes by student workers.

Within a rack we removed all stoppers and caps and gently tilted the rack to drain fluids into a collection basin, taking care not to lose specimens. Original fluids were properly disposed as hazardous chemical waste. Vials were then slowly refilled with 70% ethanol as a first rinse. The next step involved setting out 5.5 cm diameter plastic Petri dishes equivalent to the number of vials in the rack. These were placed on a 41 cm X 30.5 cm cafeteria tray labeled with the rack number and the number of vials in the rack. Unstoppered vials were inverted into Petri dish bottoms taking care to remove all contents of the vial. Vials with exterior labels were soaked in water in a second Petri dish atop the first, and the labels removed after 15 minutes. These labels were added to the first Petri dish. Vials were added to the tray in original sort order. More 70% ethanol was added to each Petri dish to cover specimens completely, and dish tops added to prevent evaporation. INHS unique identifiers (catalog numbers) consisting of a consecutive number printed on 120 g/m^2^ (32 lb) 100% white cotton, noncoated paper, were printed using an Epson WF-M1030 Series inkjet printer supplied with a 223XL cartridge. Identifiers were printed in large format (3.1 cm X 1.7 cm) to improve their visibility in the vial and to aid in maintaining a top-of-vial position. These identifiers were cut from the page and set atop each Petri dish (Fig. 2). The entire procedure was replicated with up to 10-12 racks/trays prior to imaging.

**Figure 3.**
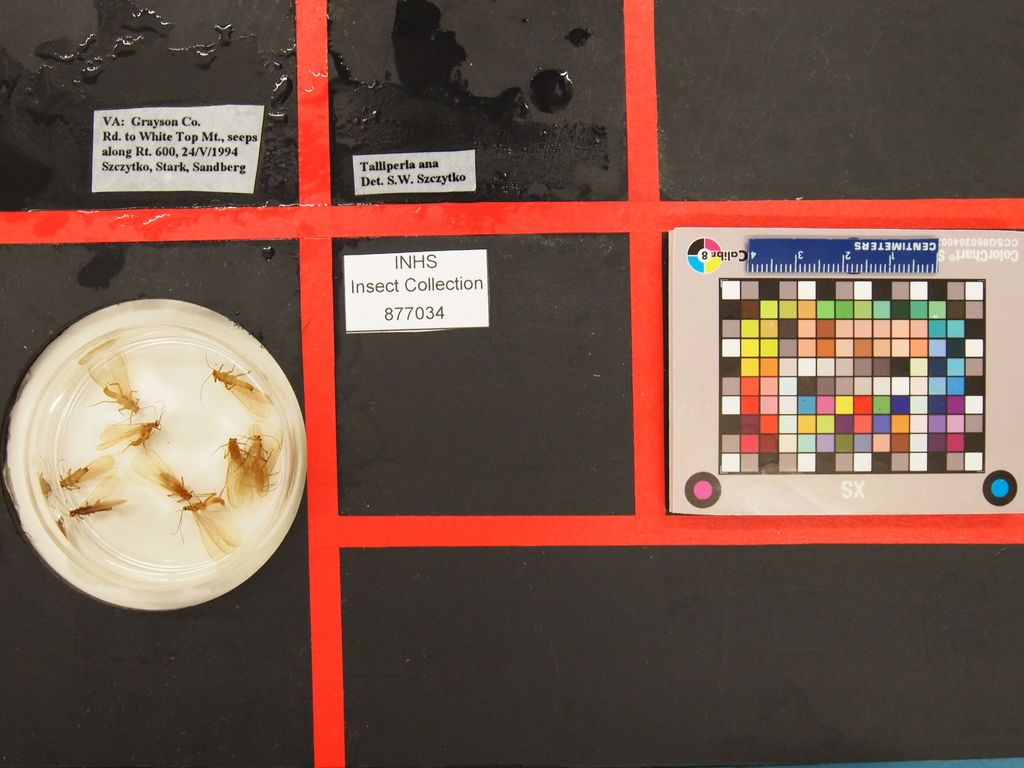
Photographic jig used for imaging Plecoptera specimens and labels. Note that labels are regionalized into particular quadrants.

**Figure 4.**
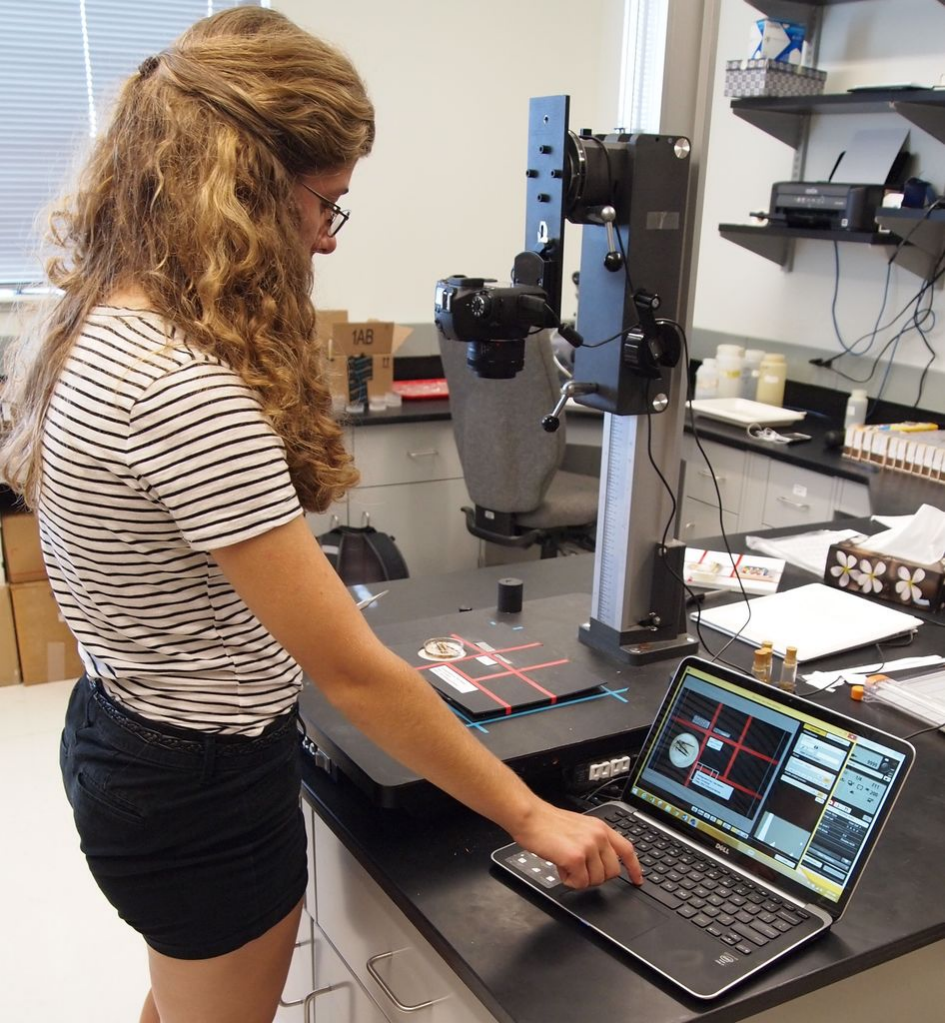
Camera, stand, and computer setup used for imaging Plecoptera specimens and labels.

**Imaging.** We built a simple photographic jig from two layers of black, high density plastic with the dimensions 25.5 cm X 20.5 cm. A well was drilled in the top layer to hold the Petri dish bottom and the two layers glued together. Red laboratory tape was used to regionalize the following quadrants: unique identifier, event/locality label(s), determination label(s), other labels, a color standard, scale, and metadata tag consisting of name of photographer, date imaged, and donor name (Fig. 3). The imaging jig layout corresponds with an image processing library ("sqed", https://github.com/SpeciesFileGroup/sqed) developed for future integration of the images into collections management software. Images of up to 20 MB were captured without flash using a Canon EOS 6D camera with a 50 mm lens, mounted on a Polaroid MP-4 photographic stand (Fig. 4). Imaging was coordinated through a Dell XPS 13 laptop equipped with EOS 6D Utility software. Images for each session were temporarily stored on this computer in a folder labelled by date and transferred to a Dropbox account for backup and sharing of images with project participants.

A Petri dish of specimens and labels was transferred to the jig, labels regionalized, and the image captured. Labels were returned to the dish, lid replaced, and catalog number placed atop the dish. If labels were printed on both sides of the label paper, these labels were fllipped and a second image taken. This dish was then moved to a new tray on the opposite side of the photographic stand. This procedure was repeated until all dishes on each tray were finished.

**Figure 5.**
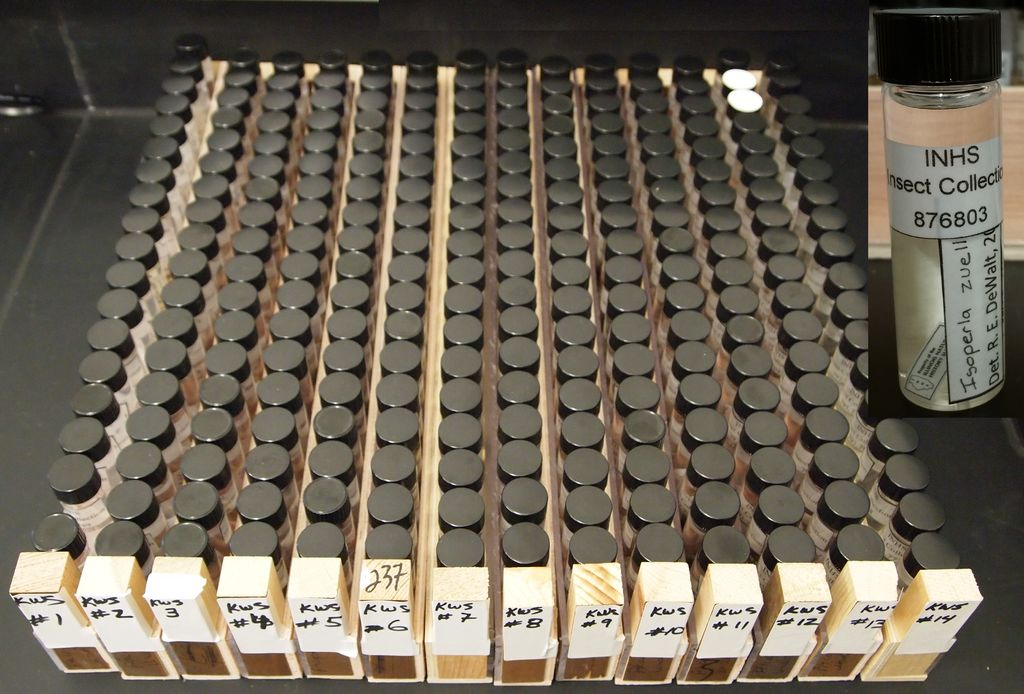
Donated Plecoptera after post-image processing in new 3 or 4 dram glass screwcap vials and racks, in original sort order.

**Post-imaging.** The original vial racks were filled with enough new 3 or 4 dram vials to match the dishes on each tray. These were prefilled with 70% ethanol. The specimens were gently added to the vials, and we attempted to arrange labels so as to be legible. The catalog number was rolled vertically and slipped into the top of the vial so that it sprang back against the glass as it was wetted. Lids were tightly screwed on and replaced in the rack (Fig. 5).

**Timing the workflow.** To determine the time necessary for each phase we timed students for 15 separate trials, each approximating about 100 vials (5 or more racks) per trial, for a total of 1,509 total vials. Mean times in decimal minutes were calculated across trials for each phase and a grand mean and standard error calculated across all trials within phases and for total time across phases. Timing in the pre-image phase did not include identification by the senior author or initial sorting of specimens by taxon into vial racks.

**Transcription of label data.** With images representing all labels and metadata, data transcription may occur without concern for re-examination of a particular vial. This allows collection managers to move specimens to long-term storage immediately after the post-imaging phase. A future article will document the refinement of another software-based transcription workflow being developed in parallel to this effort.

Data were transcribed from the images directly into Excel. Transcription of label data was iterative, yielding more specific data with each pass. Initially, only a few fields were transcribed. These included the catalog number, verbatim event label(s), verbatim determination label(s), any other labels, and the image metadata. Multiple labels of a type were separate by a verticle pipe (" | "). If no count of specimens was provided on the determination label, an actual or estimated count by stage was recorded into separate fields. Sorting of the verbatim event labels allowed grouping of labels from the same events, improving our ability to transcribe damaged labels. Subsequently, we added country, state or province, and county fields, allowing further sorting and normalization of verbatim data into fields such as locality, waterbody, public place name, dates, and collectors. Completely normalized specimen data were then imported into our INHS Insect Collection Database where georeferencing and/or linking to existing location codes took place.

**Georeferencing.** Digitization of two key documents allowed for association of many incompletely labeled specimens with sampling locations. The Bill P. Stark field notebook of 1971 and 1972 from studies on Oklahoma stoneflies (Stark and Stewart 1973a, Stark and Stewart 1973b) provided location information for several hundred vials of specimens containing minimal label data. A verbatim recording of each entry was conducted in Excel. Additional data normalized from verbatim entries included field notebook number, date of collection, state, county, locality, and public land name. A large number of Stewart specimens originated from Ozark and Ouachita Mountains work conducted by Poulton and Stewart (1991). Poulton collected thousands of specimens and the contents of many vials were labeled with only a number code corresponding to routinely visited Arkansas or Missouri locations. These codes were placed on the determination label along with a date of collection. A number alone corresponded to an Arkansas location, while one with an "M" preceding it was a Missouri location. Poulton deposited many of these incompletely labeled specimens with Stewart, but provided full event labels that could be cut out and added to vials as needed. The user was to match the site code on the determination label to the same code in the folder containing pages of labels. Most of those specimens were never fully labeled upon arrival at the INHS. An unknown number the Poulton vials were dispersed to other stonefly researchers. Poulton's labels were digitized and normalized similiar to that of the Stark field notebook.

Locations were georeferenced using Acme Mapper 2.2, all coordinates being of geodetic datum WGS84 (Acme Mapper 2018). We recorded coordinates in latitude and longitude decimal degrees to five digits and added a radius of precision in meters using the following scale: a sampling event placing a stream at a road crossing was scored a 1=10 m radius; small town or park with or without a stream name scored a 2=1000 m radius; a relatively short drainage stream within a county scored a 3=10,000 m radius; or a river that runs across an entire county or a county level record without other data scored a 4=100,000 m radius. While examining each record on Acme Mapper 2.2, stream width in meters was estimated using the scale provided and recorded as: 1=a spring/seep, 2=1-2 m wide stream, 3=3-10 m wide stream, 4=11-30 m wide stream, 5=31-60 m wide stream, 6=61 m or greater width stream, and 7=large lake. While stream drainage area is a more continuous and quantitative way of measuring waterbody size, this information is often not available for the many small streams that are common locations for stoneflies (but see USEPA 2018).

**Mapping**. Plecoptera specimen locations were imported as XY data into Esri ArcGIS 10.6.0.8321. Large scale 1:10 m cultural (4.1.0), physical (4.1.0), and Gray Earth shaded relief raster (3.2.0) layers were downloaded from Natural Earth (2018), an open data project providing free GIS data layers released into the public domain. All data layers were projected to USA Contiguous Albers Equal Area Conic USGS version (WKID: 102039). States and provinces containing Plecoptera specimens were labeled.

**Data Sharing.** None of the specimen data from these collections have ever been available to the Global Biodiversity Information Facility (GBIF) or to iDigBio. To facilitate data sharing, occurrence data were formatted as Darwin Core Archive (DwC-A) using the DwC-A Toolkit and the Occurrence extension (GBIF 2018). These data were then run through Pensoft's Integrated Publishing Toolkit with the resulting data being available as DeWalt et al. (2018a). The Stark field notebook (Suppl. material 1) and the Poulton locations (Suppl. material 2) have also been provided in Excel Spreadsheet format.

## Results

**Figure 6.**
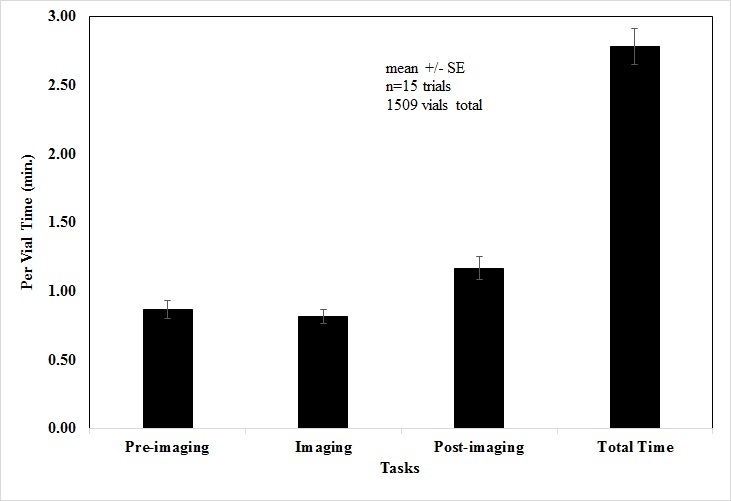
Results of time trials for pre-imaging, imaging, and post-imaging phases of accession of Plecoptera. Bars indicate mean time in minutes needed to complete each phase and the total time for all phases combined. Error bars are one standard error.

**Time to perform accession tasks.** We found that processing a single vial through pre-imaging, imaging, and post-imaging phases took on average 2.78 +/- 0.13 minutes (Fig. 6). Placing specimens and labels into new storage required the most time of the individual phases, accounting for 1.17 +/- 0.08 minutes. This is 27% higher than pre-imaging, the next most time consuming phase.

**Figure 7.**
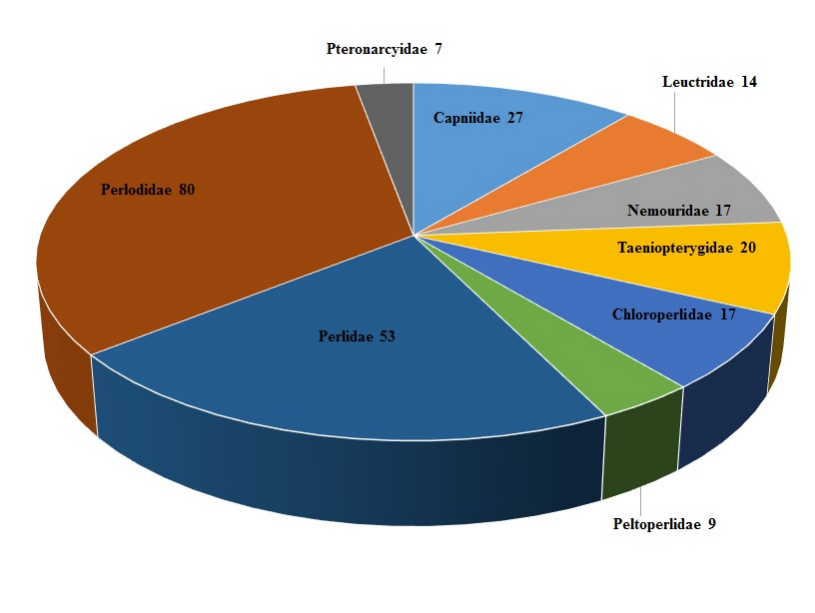
Number of stonefly species by family present in the donated Plecoptera collections. The number to the right of the family name is the number of species.

**Figure 8.**
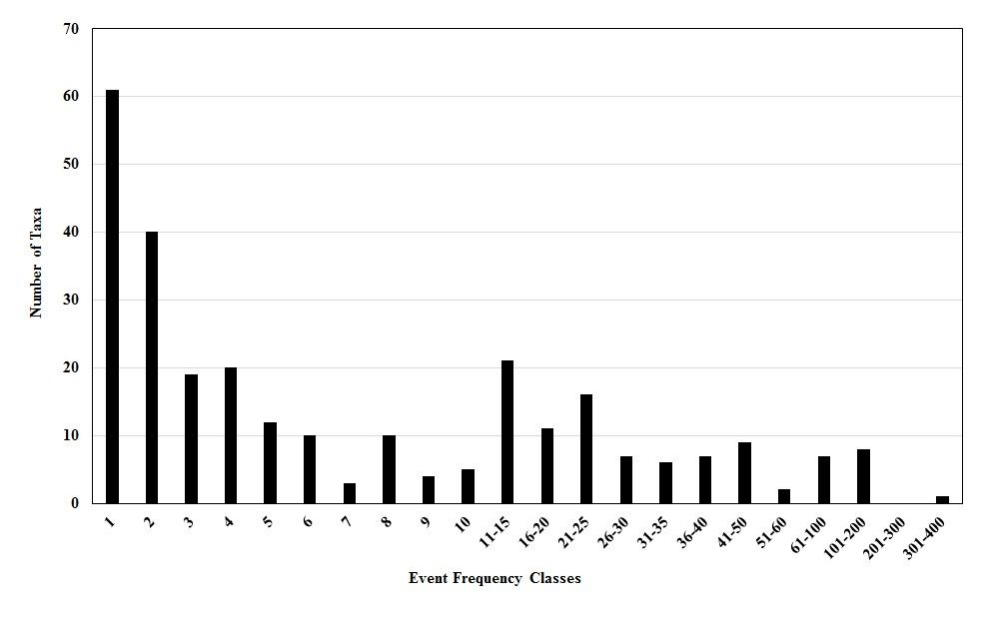
Number of stonefly taxa occurring in site/date event frequency classes.

**The Plecoptera Specimens.** A total of 5,766 specimen records resulted from the Stewart, Szczytko, and Poulton donations, constituting at least 39,968 specimens. Eleven specimen records were for mayfly (Ephemeroptera), fishfly (Megaloptera), and caddisfly (Trichoptera) taxa not treated further. A total of 243 stonefly species were recognized in the donated specimens (Table 1, Fig. 7). The greatest number of species occurred within the families Perlodidae (80 species) and Perlidae (53 species). A total of 100 rare taxa (mostly species) were represented by only one or two site/date events (Fig. 8). Ten taxa were represented by more than 100 site/date events: *Isoperla namata* Frison, 1942, *Hydroperla crosbyi* (Needham & Claassen, 1925), *Isoperla signata* (Banks, 1902), *Taeniopteryx burksi* Ricker & Ross, 1968, *Isoperla* sp., *Isoperla irregularis* (Klapalek, 1923), *Clioperla clio* (Newman, 1839), *Neoperla* sp., and *Perlesta* sp., the last being represented by 309 site/date events.

**Figure 9.**
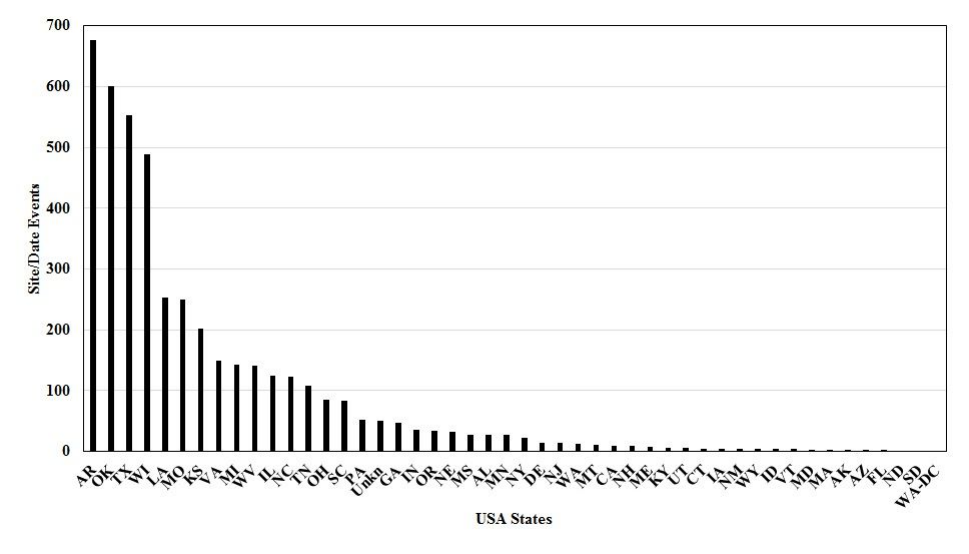
Number of site/date events for Plecoptera specimens from 48 United States and Washington D.C.

Stoneflies originated from 9 countries with the United States being represented by 48 states and the District of Columbia with 4,467 site/date events. The other countries represented were Canada with 9 provinces and territories with 51 site/date events; Mexico with 16 events; France and United Kingdom with 3 events each; and Columbia, Mongolia, Panama, and Peru each with 1 event. Within the United States, seven states were represented by 200 to 700 site/date events (Fig. 9): Arkansas, Louisiana, Missouri, and Kansas, Oklahoma, Texas, and Wisconsin.

**Figure 10.**
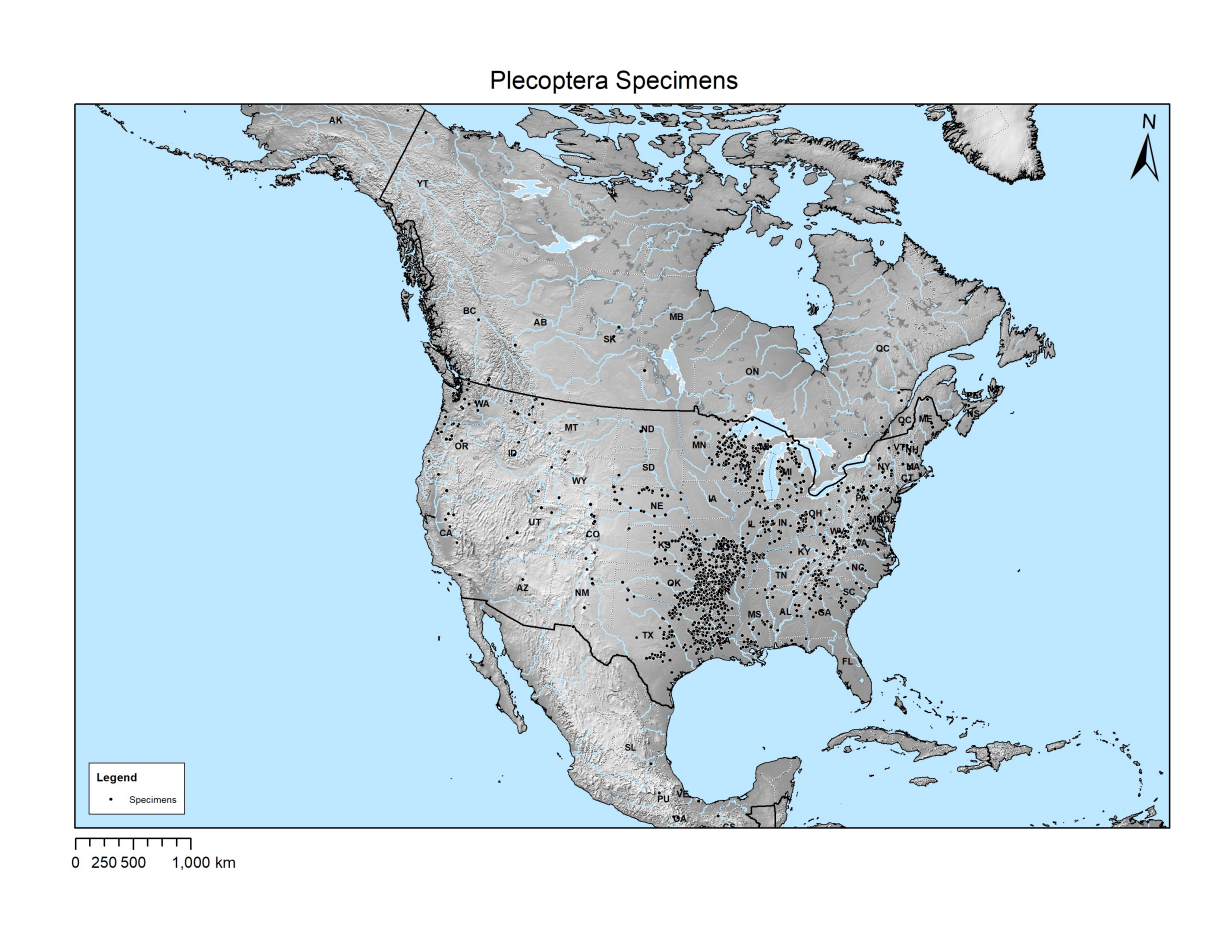
Georeferenced locations of the donated Plecoptera specimens for Canada, Mexico, and the United States.

A total of 5,633 of the 5,766 specimen records were georeferenced, the remainder had either confounded label data, were only labeled by undecipherable codes, were labeled by state only, or lacked a locality label. Mapped locations for Canada, Mexico, and the United States demonstrate three clusters of sampled locations (Fig. 10). Cluster 1 involves states within and nearby the Interior Highlands and Gulf Coastal Plains states: Arkansas, Kansas, Louisiana, Missouri, Oklahoma, and Texas. Cluster 2 encompasses the Appalachian Mountains in the east of the continent. A third cluster includes states in the middle and upper Midwest of Illinois, Indiana, Michigan, and Wisconsin.

## Discussion

### Significant findings among specimens

Most taxonomists have unfinshed business in the form of undescribed species and specimens constituting noteworthy distribution records that have never been published. Such is the case with the Stewart and Szczytko donations. We have discovered among them one new species of *Perlesta* (Perlidae) from Arkansas and a total of 21 new or confirming USA state or Canada province records (Table 1). For each record we present a brief accounting of each species including verbatim specimen event label(s), verbatim determination label(s), and unique identifier composed of collection prefix and unique number. Multiple labels for the same specimen(s) are separated from each other by " | ". Value added data, beyond that provided in verbatim records, are available in DeWalt et al. (2018a). All specimens listed here are found in the INHS Insect Collection.

**Capniidae**

***Paracapnia angulata* Hanson, 1942.** The Stewart donation yielded specimens of this species for Prince Edward Island (PEI), Canada. Stark and Baumann (2004) in their review of the genus did not report this widespread species from the province. Label data:

- PEI, 9-IV-98, Balsam Hollow Br., PEI National Park, M. Dobrin. *Paracapnia angulata*, 1 male, 1 female, Det. KW Stewart I-2001. INHS, Insect Collection 795377.

**Leuctridae**

***Leuctra ferruginea* (Walker, 1852).** The Stewart donation provided a second new province record for PEI and Illinois. This species was not previously reported from Illinois (DeWalt and Grubbs 2011), though it has been taken from Kentucky (Tarter et al. 2015), Michigan (Grubbs et al. 2012), and Wisconsin (Hilsenhoff 1995). Kondratieff and Baumann (1994) did not record this species from PEI. Label data:

- PEI, Balsam Hollow Br., PEI National Park, 25-VII-1997, M. D. Dobrin. *Leuctra ferruginea*, 5 males, 2 females, Det. KW Stewart I-2000. INHS, Insect Collection 795431.
- Illinois: Union Co., unnamed creek (flowing into Devil's Kitchen Lake), appprox. 13 mi SE of Carbondale (T11S R1E S1/2 NE 1/4 or SE 1/4) May 13, 1976, J. D. Unzicker & W. U. Brigham, blacklight (all night) J.D.U. Field Lot No. 2B. *Leuctra ferruginea*, 1 male, Det. B. C. Poulton [19]88. INHS, Insect Collection 794697. (identity confirmed by R. E. DeWalt)

***Leuctra sibley*i Claassen, 1923.** This species is also added to PEI from the Stewart collection. In Canada, this species is known from the mainland provinces of New Brunswick, Quebec, and Ontario (DeWalt et al. 2018). Label data:

- PEI, Winter Cr., Pleasant Grove, 25-VII-1997, M. D. Dobrin. *Leuctra sibleyi*, 1 female, Det. KW Stewart I-2000. INHS, Insect Collection 795436.

***Leuctra tenuis* (Pictet, 1841).** A Georgia, USA record is found for this species within the Stewart collection. It has never been reported from the state (Verdone et al. 2017), though it is known from neighboring Alabama (Harrison and Stark 2010). Label data:

- Georgia: White Co., Chattahoochee River 19-VIII-1973. *Leuctra tenuis* (Pictet) M, Det. B. Stark 1974. INHS, Insect Collection 795442.

**Nemouridae**

***Amphinemura texana*** **Baumann, 1996**. Several specimens of what was originally labeled as *A. nigritta* (Provancher, 1876) were found in the Stewart collection from southwestern Arkansas. The habitat of these specimens was similar to that reported by Baumann (1996) for neighboring Texas and Louisiana for *A. texana* Baumann, 1996. Re-examination of these specimens proved that they were indeed *A. texana*. Our data constitutes all but one of the Poulton and Stewart (1991) records for *A. nigritta*, the last one we have not seen (MO: Callaway Co., Middle R., Hwy H, E of Fullton, 30-IV-72, 1 M [male], D. A. Boehne). This calls into question the presence of *A. nigritta* in the Interior Highlands. Label data:

- #92 17-IV-85 | AR, Dallas Co., Populi Creek, 2 mi SE Forrest Bonner at Hwy 273, 17-IV-1985, B. C. Poulton. *Amphinemura texana*, 3 M, 10 N, 3 Ex., Det. R. E. DeWalt, 2018 | *Amphinemura nigritta* R 3 M, 10 N Det. B. C. Poulton | *Amphinemura* sp., good series, Det. B. C. Poulton. INHS, Insect Collection 794735.
- #54 6-IV-84 | AR, Miller Co., May Branch, 1 mi E Brightstar at Hwy 160, 6-IV-1984, B. C. Poulton. *Amphinemura texana*, 2M, 12F, 3N, 16 Exv., Det. R. E. DeWalt, 2018 | Amphinemura nigritta R 4 M, R 14 F, 3 N Det. B. C. Poulton. INHS, Insect Collection 794734.

***Shipsa rotunda* (Claassen, 1923).** The Stewart collection provided a record of this species from North Carolina. Though these were only nymphs, they were with a doubt this species. They have not been previously reported from North Carolina (DeWalt et al. 2018). Label data:

- North Carolina, Davie Co., Yadkin R., 5A-04-a (S-7), 6-II-1974, R. L. Newell. *Shipsa rotunda* nymph Det. [R.W.] Baumann [19]'74. INHS, Insect Collection 795540.

**Taeniopterygidae**

***Taeniopteryx nivalis* (Fitch, 1847).** The Szczytko collection produced this species from his boyhood state of New Jersey. To date, only three species of *Taeniopteryx* have been reported from New Jersey (Earle 2009).

- NJ. S. Branch Raritan R. 28/I/89 J. Kurtenbach. *Taeniopteryx nivalis*, 7 N (larvae), Det. R. E. DeWalt, 2018 | *Taeniopteryx nivalis* [7 N] Det. S. W. Szczytko 1989. INHS, Insect Collection 877016.

***Taeniopteryx parvula* Banks, 1918.** The Stewart collection yielded this species from southeastern Oklahoma. Stark and Stewart (1973b) did not list T. parvula from Oklahoma.

- Clear Creek, Choctaw Co. Okla. Jan. 31, 1972 Stark 12-7. *Taeniopteryx parvula*, F [female], Det. R. E. DeWalt, 2018 | *Taeniopteryx parvula* 1 F Det. K. E. Fullington 1978 | *Taeniopteryx* 1 F reared Det. Stark. INHS, Insect Collection 795921.

**Perlidae**

***Acroneuria evoluta* Klapalek, 1909.** Szczytko and Stewart (1977) published the definitive treatment of Texas stoneflies. In their treatment they reported this species as *A. mela* Frison, 1942 from two locations (Montgomery and Nacogdoches counties of east Texas). Those working from the old list may not know that this name is now a junior synonym of *A. evoluta* (Stark and Brown 1991). Prior to Stewart's death in 2012, he had gathered material for an update of the Texas fauna. Among this material were five series of *A. evoluta*, all from new locations. Label data:

- Texas: Polk Co. Big Sandy Cr @ Sunflower Rd. Dallardsville 4 mi W 26.IV.1996 J. C. Abbott #472, uv. *Acroneuria evoluta* 1 F Det. KW Stewart IX-1996 | *Acroneuria mela* Frison 1 F Det. K. D. Alexander 1996. INHS, Insect Collection 795000.
- Texas: Angelina Co. White Oak Cr. @ hwy 59 Lufkin, 12 mi S 7-V-1994 J. W. Chirhart uv light. *Acroneuria evoluta* 2 M Det. KW Stewart IX-1996 | *Acroneuria evoluta* Klapalek 2 M Det. K. D. Alexander 1996. INHS, Insect Collection 795003.
- SRM93-06 Texas: Hardin Co., Turkey Creek, ca. 4 mi E US Hwy 287/69, ca. 8 mi N Kountze 17-V-1993 SR Moulton. *Acroneuria evoluta* 5 F Det. KW Stewart IX-1996. INHS, Insect Collection 795002.
- Plainview Hale Co., Tex. VI-28-67 DAO. *Acroneuria evoluta*, 3 M, Det. R. E. DeWalt, 2014 | *Acroneuria mela* Frison 3 M, Det. B. Stark 1974 | *Acroneuria arida* (Hagen) [Jewett] 1968. INHS, Insect Collection 796923.
- Texas: Angelina Co. Boykin Springs @ Boykin Spr. Campground; Jaspers, 22 mi NW 6.V.1995 J.C. Abbott #305 & K. Moore, uv. *Acroneuria evoluta*, 3 M, Det. R. E. DeWalt, 2018 | *Acroneuria evoluta*? 3 M Det. KW Stewart IX-1996 | *Acroneuria mela* 3 M Det. K. D. Alexander 1995. INHS, Insect Collection 795001.

***Acroneuria frisoni* Stark & Brown, 1991.** This species is a new state record for Texas. The name has a complex history that is explained in Stark and Brown (1991). Throughout much of the 20th century, this species was erroneously referred to as *A. evoluta*. Under that name, it had been reported from Oklahoma (Stark and Stewart (1973b) and the Interior Highlands (Ozark and Ouachita Mountains) of Arkansas and Missouri (Poulton and Stewart 1991), where it is common. The species has a wide distribution from the Ozark Mountains eastward and as far north as southern Ontario (Cao et al. 2013, Pessino et al. 2014). The Stewart collection provides records from two locations in two counties--contrary to verbatim labels, all Boykin Springs collections were taken in Jasper County.

- SRM93-05 TX: Hardin Co. Hickory Creek, ca. 1.5 mi E. Hwy 287/69, ca. 8 mi N. Kountze Tx 17-V-93 Moulton. *Acroneuria frisoni* , 1 M, Det. KW Stewart IX-1996. *Acroneuria frisoni* , 1 M, Det. KW Stewart IX-1996. INHS, Insect Collection 795005.
- TX: Jasper Co. Boykin Sprgs. Angelina N.F. 20 mi NW Jasper 13-VI-94, J. Abbott, J. Chirhart, M. Passante #199 UV. *Acroneuria frisoni* , 2 M, Det. KW Stewart IX-1996. INHS, Insect Collection 795004.
- TX Angelina Co. Boykin Sprgs. @ Campground Jasper 22 mi N/W 6-V-1995 Abbott & Moore UV lights #305. *Acroneuria frisoni*, 3 M, Det. KW Stewart IX-1996 | *Acroneuria frisoni* Stark & Brown 3 M Det. K. D. Alexander 1996. INHS, Insect Collection 795007.
- TX Angelina Co. Boykin Sprgs. @ Campground Jasper 22 mi N/W 6-V-1995 Abbott & Moore UV lights #305. *Acroneuria frisoni*, 2 M, 1 F, Det. KW Stewart IX-1996. INHS, Insect Collection 795006.

***Agnetina flavescens*** (Walsh, 1862). The genus *Agnetina* and its three species Nearctic species have been confused for most of the 20th century until Stark (1986) revised the genus. No member of the genus has ever been reported from Texas. The Stewart material provides the first specimen, constituting a new generic record and extension of *A. flavescens* into Texas. This species is known from Oklahoma (Stark 1986), the Ozark and Ouachita Mountains (Poulton and Stewart 1991), and most states eastward to the Atlantic Coast (DeWalt et al. 2018).

- Plainview, Hale Co., Tex. VI-28-67 DAO. *Agnetina flavescens*, N Det. R. E. DeWalt, 2014 | *Phasganophora capitata* (Pictet) nymph Det. B. Stark 1994. INHS, Insect Collection 794060.

***Neoperla occipitalis* (Pictet, 1841)**. The Szczytko collection provides a series of this species from the Upper Peninsula of the Michigan, constituting an new state record for Michigan. Its presence in Michigan is not surprising since it has been reported from Illinois and Indiana (DeWalt and Grubbs 2011), Ohio (DeWalt et al. 2016), Ontario (Stark and Baumann 1978), and Wisconsin (Stark 2004).

- MI, Dick[in]son Co. Sturgeon R. Hwy 2, 4 mi E. Iron Mt. 27/VII/1989 S. W. Szczytko. *Neoperla occipitalis* (Pictet) [7 ad.] Det. B. Stark 1989. INHS, Insect Collection 876842.

***Perlesta* AR-1 n. sp.** The new species is currently being described and has been identified from several locations in Arkansas from the Stewart specimens. It has also been found to be relatively common in eastern Oklahoma from Oklahoma State University material currently being examined. We refrain from providing detailed location information at this time.

***Perlesta shubuta* Stark, 1989**. This Gulf Coastal Plains species has been confused with a recently described species, *P. ephelida* Grubbs & DeWalt, 2012, so records older than 2012 must not be accepted at face value. So far, the only confirmed records of this species are from Alabama, Florida, Louisiana, and Mississippi (Graves and Ward 2011, Grubbs and DeWalt 2012, Stark 1989). The Stewart collection provides a new state record for Missouri. Label data:

- M67 24-V-86 [a coded location for the following BCPoulton MO-67, Missouri, Dallas Co., 1 mi E Buffalo at Hwy 32, 24-V-1986, B. C. Poulton]. *Perlesta shubuta*, M, Det. R. E. DeWalt, 2015. INHS, Insect Collection 793694.

***Isoperla montana* (Banks, 1898)**. Until the recent treatment of eastern North American Isoperlinae (Szczytko and Kondratieff 2015), this species was difficult to consistently discern from *Isoperla namata* Frison, 1942 and an undescribed species common in the eastern USA now known as *I. kirchneri* Szczytko & Kondratieff, 2015. The Szczytko collection yielded specimens from Massachusetts, Oklahoma, and Vermont, all new state records. It is now known from much of the eastern USA and eastern Canada (DeWalt et al. 2018b, DeWalt et al. 2018, Szczytko and Kondratieff 2015). Label data:

- Ware Center, Mass., 21 May 1938, Col. J. F. Hanson. *Isoperla montana*, M, F, Det. R. E. DeWalt, 2018 | *Isoperla montana* (Banks) M, F, Det. J. F. Hanson. INHS, Insect Collection 877158.
- Okla. Delaware Co. Flint Crk., Hwy 33, 21-IV-79 S. W. Szczytko, K. W. Stewart, B. P. Stark. *Isoperla montana*, F. Det. R. E. DeWalt, 2018 | *Isoperla montana* 1 F Det. S. W. Szczytko. INHS, Insect Collection 876154.
- Hitchcock's Brook Pittsford Vt. V-20-66 J. W. Hitchcock. *Isoperla montana*, M, 2 F, Det. R. E. DeWalt, 2018 | M, 2 F *I. signata*?. INHS, Insect Collection 876450.

***Isoperla signata* (Banks, 1902)**. The Szczytko collection yielded this species from New Hampshire, a new state record. The species is known from nearly all states and provinces from Oklahoma and Manitoba eastward, except the Gulf Coastal Plains states (Szczytko and Kondratieff 2015).

- USA: NH: Strafford Co. Lamprey River, Packers Falls, 3 km SW Durham, VI-8-2009 D. S. Chandler. *Isoperla signata*, F, Det. R. E. DeWalt, 2018 | *Isoperla richardsoni* [D. S. Chandler]. INHS, Insect Collection 876429.

***Isoperla similis* (Hagen, 1861).** Szczytko and Kondratieff (2015) considered the concept of this species to only include specimens from Maryland, New Hampshire, New Jersey, New York, Pennsylvania, and Virginia. Included among the Szczytko collection were two females of this species from Vermont, a new state record. Label data:

- Jacksonville Vt. June 20, 1937 Col. J. F. Hanson. *Isoperla similis*, 2 F, Det. S. W. Szxzytko, 1990 | *Clioperla similis* (Hagen) F, Det. J. F. H. Feb. 5, 1941. INHS, Insect Collection 877188.

***Isoperla zuelligi* Szczytko & Kondratieff, 2015.** This species was originally described from North Carolina (Szczytko and Kondratieff 2015) and was recently reported by Grubbs and Sheldon (2018) from several locations in Alabama. The Szczytko collection yielded one female specimen from New Hampshire, a tremendous range extension. This female specimen agrees in all respects with the original description, most importantly in its unique egg ultrastructure: the posterior pole with a low collar and elongate anchor and well developed follicle cell impressions having wide margins. In this specimen, eggs also have a mushroom shaped, membranous cap as confirmed in Grubbs (2016) by scanning electron microscopy (SEM) of Alabama specimens. Our examination using light microscopy revealed that the spindle is longer than depicted previously and that removal of the cap for SEM reduced the spindle length. We found the cap to be studded with papillae.

Another specimen from the Szczytko collection was labeled as holotype for "*Isoperla grahami*", a manuscript specimen resulting from the James (1972) dissertation on the stoneflies of Alabama. It was never described and the name is not valid. In consultation with Scott Grubbs (Western Kentucky University) and through examination of the specimen, it was found to be to I. *zeulligi* Szczytko & Kondratieff, 2015. We report it here in light of it being a manuscript type from the James (1972) dissertation. Label data:

- USA: NH: Strafford Co. Lamprey River, Packers Falls, 3 km SW Durham, VI-8-2009, D. S. Chandler. *Isoperla zuelligi*, F, Det. R. E. DeWalt, 2018 | *Isoperla richardsoni* [Det. S. Chandler]. INHS, Insect Collection 876429.
- Dekalb Co., Ala. Powell, Ala. Creek S. of light on Ala. 35. N. of Rainsville May 18, 1972 A. James & A. Burnett. *Isoperla zuelligi*, M, Det. R. E. DeWalt, 2018 | *Isoperla grahami* n.s. male holotype det. A. James 1972. INHS, Insect Collection 876803.

### Workflow and disruptions

Large donations of wet collections pose many problems for accession. Often they require much handling of specimens to accomplish all necessary tasks, and these activities risk damage to the specimens. An efficient workflow that minimizes specimen handling would help to prevent damage. Our workflow accomplished multiple tasks at one time: it removed specimens from old storage, removed and rinsed old preservatives, assigned unique identifiers to each unit, imaged the specimens and labels, moved specimens to new storage, and transcribed the label data. The average time to move a vial across preimaging, imaging, and postimating phases was under 3 minutes, and under some cirucumstances, could be much shorter. Because we used Petri dishes to hold the contents of the original vials, the process and timing could be stopped at anytime and resumed again, even one to two days later, as long as enough ethanol was present in the dish and a lid applied. It was important for us to think "industrial" in order to gain efficiencies of scale. Similar tasks were grouped and done in large numbers to make the task efficient. It was always worth asking "How do we tackle more vials at once?"

It is our experience that most undergraduate students do not enter a laboratory with the mindset to develop more efficient workflows for assigned tasks. Do not assume that your students, or even a coworker, looks for efficiences. We had to help them develop this philosophy by demonstrating that grouping like tasks together, setting goals for completion, and timing each phase of the process yields a superior product, yet does so with less overall time spent. We provided students with written instructions, templates for producing metadata labels, standardized data sheets for recording their name, total number of racks, vials per rack, and begin and end times for each particular phase. We walked them through each step of the process several times with small sets of specimens until they got used to the procedure. We then forced them to stretch their abilities by adding several more vial racks and vials until they could process 10-12 racks, each containing up to 21 vials. We insisted that students worked blocks of time sufficient to complete at least one of the phases of the workflow. There is no doubt that this exercise was illuminating for most students; therefore, we believe that the experience will serve them well in the future.

It is worth discussing some difficulties that slowed our workflow. Many specimens were stored in patent-lip vials with failing stoppers. Often, the stoppers were so swollen that their removal could only be done in pieces. We resorted to using inexpensive glass tube cutters to safely remove the tops of vials and stoppers. Opening vials in this manner normally added 30 seconds to the pre-imaging phase. Our collection, and others, have found the task of purchasing archival quality stoppers for patent-lip vials to be impossible. Stoppers that are currently available tend to swell in preservative, harden, and shrink at the top, allowing for evaporation. This is our reasoning for going to screwcap vials with beveled plastic caps for most wet insect specimens.

Additional difficulties arose from the 10-15% of vials that had external labels. Most of these were our own INHS specimens borrowed decades ago by Szczytko. Many of these labels had been tightly adhered to the vials for 70-80 years! Soaking off the label generally required 15 minutes in water, but in reality added little time to the procedure since the soaking took place in a second Petri dish atop the first. Internal labels were frequently more problematic. Some colleagues coil long, thin labels atop the vial. This placement helped the donors read locations and determinations quickly, but removal of such a label is difficult without damaging it, and putting them back in is even more frustrating. These were pulled out, flattened for imaging, and often recut for vertical placement in the vial. This was necessary since the coil replaced in the vial rarely stays atop the vial. Extra large, often folded labels were often worse, forcing students to gently remove them from the vial, unfold them, flatten them for imaging, and refold them for placement in the new vial. We believe in this case that a new label should be written in smaller format for inclusion with the original label.

Our experience with laser printed and photocopied labels has demonstrated that at least older ones were not of archival quality. This conclusion is based on examination of nearly 6,000 sets of labels. We routinely found labels where letters were sloughing off the paper, and in the case of photocopied labels, careless handling could smudged the entire label. Please take care when handling old labels. The images we captured preserved what information remained and iterative transcription and sorting grouped damaged and undamaged labels from the same event, aiding in recovery of information.

To ensure the longevity of labels, it is important to avoid adopting new practices that have not been time-tested. We suggest that no laser or other toner based labels be used for wet specimens. Even under the best of conditions, toner of laser printed labels often chatters from letters near cut edges and abraids easily when being gripped with forceps, when slid past openings in vials, or when rubbed against other labels. Be aware that stacking of anything on sheets of laser printed labels immediately begins abrasion. For mass produced labels, an ink jet printer with indelible ink seems be the best alternative. Otherwise, labels should be written by hand using an alcohol fast pen such as a Pigma Micron_TM_.

For standard vials (3 or 4 dram), labels should be made a little longer than wide and long enough that when slid in lengthwise, they stand upright in the vial for easy reading. Labels should not be coiled because it makes imaging labels and upgrading storage in the future more difficult. If using printed catalog numbers, print them on moderately heavy (32 to 36 lb) archival paper in a format wide enough that when added to a vial the label will spring back against the glass and will be held in place, preferably at the top of the vial.

A recent paper by Mendez et al. (2018) has some relevance for this study. They created a photographic jig, using 3D printing, to image either dry or wet specimens and labels. They tested several colors and plastics formulations, finding that some combinations were better than others. Unfortunately, they did not conduct any time trials, so we cannot make comparisons with our efforts. However, adoption of 3D printing of our jig would greatly increase its efficiency and precision of production. We would be able to optomize through testing of various backgrounds and regionalizing colors. This work would help to us create sharper, more contrasting images that would improve the performance of our new software for reading text from images.

### Importance of data set

The specimen data resulting from the accession of these Plecoptera donations have never been available electronically. Major works that provided specimens in these donations include Stark and Stewart (1973a) and Stark and Stewart (1973b) for Oklahoma, Szczytko and Stewart (1977) for Texas, Stewart et al. (1976) for Louisiana, Poulton and Stewart (1991) for the Ozark and Ouachita Mountains of Arkansas and Missouri, Stewart and Stark (1988) and Stewart and Stark (2002) for all of North America, and Szczytko and Stewart (1979) and Szczytko and Kondratieff (2015) for *Isoperla*. Many other taxonomic and distributional works report specimens contained in these donations, but are too numerous to cite here.

Stoneflies are susceptible to relatively small changes in water and habitat quality. Agriculture and urbanization have extirpated 20 Illinois stonefly species, some of which were once widespread and abundant (DeWalt et al. 2005). Bojkova et al. (2012) have noted similar dramatic losses for fixed sites in the Czech Republic. Giersch et al. (2015) have documented range reduction of an alpine stonefly, *Zapada glacier* (Baumann & Gaufin, 1971), due to climate change related shrinking of glaciers. Sheldon (2012) reported shifts in altitudinal zonation for stonefly species in the Great Smoky Mountains National Park in the eastern states of Tennessee and North Carolina, presumably the result of climate change. Given their sensitivity, we need to gather all the verified stonefly specimen information from institutional and private collections to help us define their historical distribution, advocate for their conservation, and predict the effects of multiple stessors in the present and future.

### Future work involving these specimens

The specimens and their data are now well protected. Most are identified to species, but still hundreds of vials contain specimens that are identified only to genus. Some specimens are larvae with little hope of further identification, but others are adults where further identification is possible. The most important adults are the small Perlidae stoneflies *Neoperla* (166 site/date events) and *Perlesta* (309 site/date events) and Perlodidae in the genus *Isoperla* (136 site/date events). Recent works have now made identification of adult specimens in these genera possible (Stark 2004, Szczytko and Kondratieff 2015) and this may be accomplished even if specimens are not quite in perfect condition. The reader is invited to borrow these specimens for study.

Some studies conducted in Texas and surrounding states need to be replicated and the hundreds of specimens in these donations should form the basis for such studies. The Kansas (Stewart and Huggins 1977), Louisiana (Stewart et al. 1976), Oklahoma (Stark and Stewart 1973a, Stark and Stewart 1973b), and Texas (Szczytko and Stewart 1977) studies would benefit by updating what is known about each state's small perlids, e.g., *Perlesta* and *Neoperla*. In these studies, only *Perlesta placida* (Hagen, 1861) and *Neoperla clymene* (Newman, 1839) were listed. At time of publication, these were so-called "trash can" species, serving as names for many species unrecognized at the time. A revolution in the taxonomy of these genera has occurred since then, much of which is summarized in Stark (2004). Even Arkansas and Missouri, last studied by Poulton and Stewart (1991) require revision. This study placed many species in watersheds and identified factors important in governing watershed affiliation. The key was good work, but is becoming outdated. Unfortunately, almost no specimen data were provided in the paper. No secondary objectives from this major work can be accomplished due to the lack of easily accessible specimen data. It is our hope that new studies would adopt modern standards of biodiversity research: digitization of the specimen data, assigning a unique identifier to each unit (vial or pin), and sharing of the data in human and machine readable formats. Meeting these criteria improves data sharing and use of the data for secondary objectives such as conservation, modeling of distributions, and easy comparisons with other time frames. DeWalt and colleagues are in the process of updating the Oklahoma stoneflies, having borrowed material from Sam Noble Museum in Normal and the Oklahoma State University Insect Collection in Stillwater. We invite others to borrow material for other states and provinces.

## Conclusions

We have demonstrated an efficient workflow for accessioning wet insect collections that combines transfer to new storage, imaging of specimens and labels, and transcription of the data. Images largely eliminated the problem of verification of transcribed text against a verbatim source. Our iterative approach to transcription has advantages in that it allows for sorting after minimal transcription, resulting in the pairing of like labels and focused normalization of one or a few data types at a time.

We have protected the specimen legacy of important stonefly researchers through our efforts. The specimens are stabilized, the nomenclature and many identifications updated, and all data available digitally and shared globally (DeWalt et al. 2018a). We have provided access to a field notebook for Oklahoma specimens (Suppl. material 1) and a document that links coded locations to hundreds of vials of specimens from Arkansas and Missouri (Suppl. material 2). Re-interpretations of specimens may be updated easily in the future by matching the unique identifiers and digital data for specimens. Finally, it is possible now for local, state, regional, and federal agencies to access the data to meet additional objectives.

## Supplementary Material

Supplementary material 1 B.P. Stark 1971/1972 Digitized Field Notebook for Plecoptera Collected in Oklahoma. Data type: Site/date events tied to unique locations. Excel sheet with multiple worksheets.
Brief description: B.P. Stark 1971/1972 Digitized Field Notebook for Plecoptera Collected in Oklahoma. Site/date events are codes are linked to unique locations among the Stewart specimens. The format is an Excel_TM_ sheet with multiple worksheets.
File: oo_231194.xlsx RE DeWalt, EA Snyder

Supplementary material 2 Key to Poulton Coded Locality Codes in Stewart, Szczytko, and Poulton Collections from Arkansas and Missouri. Data type: Locations for occurrences. ExcelTM spreadsheet with two worksheets.
Brief description: Poulton provided coded locations for hundreds of vials of stoneflies collected in Arksansas and Missouri contained within the Stewart, Szczytko, and Poulton collections. Some additional vials remain scattered among other institutions (Brigham Young University, Colorado State University and others). This file will help to link coded specimen data to the Poulton locations.
File: oo_231198.xlsx RE DeWalt, E Snyder

## Figures and Tables

**Table 1.** Taxa resulting from accession of the K. W. Stewart, S. W. Szczytko, and a portion of the B. C. Poulton donations into the INHS Insect Collection. Events represent the number of unique site/date visits per species that are present in the data set. Countries are spelled out completely with the exception of the United States, which is represented by the acronym "USA". ISO 3166-2 alpha-2 codes are presented for states, provinces, and territories of Canada and the United States (https://en.wikipedia.org/wiki/ISO_3166-2). Subdivisions of other countries are spelled out. New state, province, or territory and some confirming records are represented by "*" next to the subdivision name or abbreviation. Taxon name spelling, authority, and year are from Plecoptera Species File (DeWalt et al. 2018). Those taxa listed by generic name only may be larvae or adults where further identification is possible. See detailed occurrence data in (DeWalt et al. 2018a).

**Taxon**	**Events**	**Distribution**
** EPHEMEROPTERA **		
** Ephemerellidae **		
* Drunella *	1	USA: OR
* Ephemerella *	2	USA: OK
** Heptageniidae **		
* Epeorus *	1	USA: VA
*Maccaffertium pudicum* (Hagen, 1861)	1	USA: VA
*Raptoheptagenia cruentata* (Walsh, 1863)	1	USA: IA
** MEGALOPTERA **		
** Corydalidae **		
*Chauliodes rastinicornis* Rambur, 1842	1	USA: WI
** PLECOPTERA **		
** Capniidae **		
*Allocapnia aurora* Ricker, 1952	1	USA: TN
*Allocapnia curiosa* Frison, 1942	1	USA: WV
*Allocapnia forbesi* Frison, 1929	3	USA: OH, VA
*Allocapnia granulata* (Claassen, 1924)	90	USA: AR, LA, MO, OK, VA, WI
*Allocapnia harperi* Kirchner, 1980	1	USA: VA
*Allocapnia illinoensis* Frison, 1935	2	USA: WI
*Allocapnia jeanae* Ross, 1964	2	USA: AR, MO
*Allocapnia malverna* Ross, 1964	49	USA: AR, LA, TX
*Allocapnia minima* (Newport, 1851)	5	USA: WI
*Allocapnia mohri* Ross & Ricker, 1964	60	USA: AR, MO, OK
*Allocapnia mystica* Frison, 1929	3	USA: AR, MO
*Allocapnia nivicola* (Fitch, 1847)	6	USA: VA, WV
*Allocapnia ozarkana* Ross, 1964	1	USA: AR
*Allocapnia peltoides* Ross & Ricker, 1964	1	USA: AR
*Allocapnia pygmaea* (Burmeister, 1839)	4	USA: NY, WI
*Allocapnia recta* (Claassen, 1924)	10	USA: TN, VA, WV, WI
*Allocapnia rickeri* Frison, 1929	36	USA: AR, MO, OK, VA, WV
*Allocapnia sandersoni* Ricker, 1952	4	USA: AR, MO
*Allocapnia stannardi* Ross, 1964	2	USA: NC
*Allocapnia virginiana* Frison, 1942	1	USA: NC
*Allocapnia vivipara* (Claassen, 1924)	26	USA: AR, KS, MO, OK, WI,
*Allocapnia zola* Ricker, 1952	1	USA: WV
* Allocapnia *	43	USA: AR, KS, LA, MO, NJ, OK, TN, TX, VA, WV, WI
*Isocapnia integra* Hanson, 1943	1	USA: OR
* Isocapnia *	1	USA: no data
*Mesocapnia frisoni* (Baumann & Gaufin, 1970)	5	USA: KS, TX
*Nemocapnia carolina* Banks, 1938	2	USA: AR, SC
*Paracapnia angulata* Hanson, 1942	21	Canada: ON, PE*. USA: AR, MI, MO, NC, OK, VA, WI, WV
*Paracapnia opis* (Newman, 1839)	2	USA: WI
* Paracapnia *	1	USA: WI
** Leuctridae **		
*Leuctra duplicata* Claassen, 1923	3	Canada: ON, PE
*Leuctra ferruginea* (Walker, 1852)	6	Canada: NS, PE*. USA: IL*
*Leuctra moha* Ricker, 1952	1	USA: LA--a female, uncertain
*Leuctra sibleyi* Claassen, 1923	4	Canada: PE*. USA: WI, WV
*Leuctra tenuis* (Pictet, 1841)	19	USA: AR, GA*, MO, OH, OK, WI
* Leuctra *	6	Canada: NS. USA: AR, GA, TN, WV
*Paraleuctra sara* (Claassen, 1937)	2	Canada: QC. USA: GA
*Zealeuctra arnoldi* Ricker & Ross, 1969	10	USA: TX
*Zealeuctra cherokee* Stark & Stewart, 1973	4	USA: AR, OK
*Zealeuctra claasseni* (Frison, 1929)	47	USA: AR, KS, OH, OK, TX
*Zealeuctra hitei* Ricker & Ross, 1969	33	USA: TX
*Zealeuctra narfi* Ricker & Ross, 1969	9	USA: AR, WI
*Zealeuctra stewarti* Kondratieff & Zuellig 2004	2	USA: TX
*Zealeuctra wachita* Ricker & Ross, 1969	1	USA: AR
*Zealeuctra warreni* Ricker & Ross, 1969	23	USA: AR, OK
* Zealeuctra *	5	USA: OK, TX
** Nemouridae **	4	USA: AR, OK
*Amphinemura delosa* (Ricker, 1952)	25	USA: AR, MO, OK, WI
*Amphinemura nigritta* (Provancher, 1876)	2	Canada: PE. USA: VA
*Amphinemura palmeni* (Koponen, 1917)	1	USA: WI
*Amphinemura texana* Baumann, 1996	15	USA: AR*, LA, TX
*Amphinemura varshava* (Ricker, 1952)	2	USA: WI
*Amphinemura wui* Claassen, 1923	7	USA: NC, TN, VA
* Amphinemura *	27	Canada: PE. USA: AL, AR, KS, LA, NC, OK, TN, TX, VA, WI
*Nemoura arctica arctica* Esben-Petersen, 1910	8	Canada: ON. USA: AK, NH, WI
*Ostrocerca albidipennis* (Walker, 1852)	2	USA: VA
*Ostrocerca complexa* (Claassen, 1937)	1	USA: WV
*Ostrocerca truncata* (Claassen, 1923)	2	USA: NY, VA
*Paranemoura perfecta* (Walker, 1852)	5	USA: NH, VA
*Prostoia completa* (Walker, 1852)	15	USA: MN, SC, VA, WI
*Prostoia ozarkensis* Baumann & Grubbs 2014	12	USA: AR, MO, OK
*Prostoia similis* (Hagen, 1861)	8	USA: MO, WI, WV
*Shipsa rotunda* (Claassen, 1923)	6	USA: MI, NC*, VA, WI
*Soyedina carolinensis* (Claassen, 1923)	1	USA: VA
*Soyedina vallicularia* (Wu, 1923)	2	USA: MA, VA
* Soyedina *	1	USA: NC
** Taeniopterygidae **	1	USA: VA
*Bolotoperla rossi* (Frison, 1942)	5	USA: VA
*Oemopteryx contorta* (Needham & Claassen, 1925)	11	USA: NH, TN, VA, WV
*Oemopteryx glacialis* (Newport, 1848)	13	USA: MI, WI, WV
*Strophopteryx appalachia* Ricker & Ross, 1975	4	USA: VA, WV
*Strophopteryx arkansae* Ricker & Ross, 1975	10	USA: AR
*Strophopteryx cucullata* Frison, 1934	31	USA: AR, OK
*Strophopteryx fasciata* (Burmeister, 1839)	49	USA: AR, MO, NJ, NY, OH, OK, PA, VA, WI, WV
*Strophopteryx limata* (Frison, 1942)	2	USA: TN, VA
* Strophopteryx *	19	USA: KS, NC, OK, TN, WV
*Taenionema atlanticum* Ricker & Ross, 1975	8	USA: NH, PA, TN, VA, WV
*Taeniopteryx burksi* Ricker & Ross, 1968	124	USA: AR, IL, IN, KS, LA, MI, MS, MO, NE, OH, OK, PA, TN, TX, VA, WI, WV
*Taeniopteryx lita* Frison, 1942	16	USA: IL, IN, LA, SC, TX, WV
*Taeniopteryx lonicera* Ricker & Ross, 1968	14	USA: AR, LA, MS, NJ, NC, TX
*Taeniopteryx maura* (Pictet, 1841)	40	USA: AR, GA, MD, MS, MO, NY, NC, OK, PA, TN, TX, VA, WV
*Taeniopteryx metequi* Ricker & Ross, 1968	22	USA: AR, MO, OH, VA, WV
*Taeniopteryx nelsoni* Kondratieff & Kirchner, 1982	3	USA: VA
Taeniopteryx nivalis (Fitch, 1847)	30	Canada: ON. USA: IL, MI, MN, NJ*, NY, WI
*Taeniopteryx parvula* Banks, 1918	25	Canada: QC. USA: AR, GA, IL, MI, NM, NY, OK*, PA, VA, WI
*Taeniopteryx robinae* Kondratieff & Kirchner, 1984	1	USA: SC
*Taeniopteryx starki* Stewart & Szczytko, 1974	6	USA: TX
*Taeniopteryx ugola* Ricker & Ross, 1968	11	USA: TN, VA, WV
* Taeniopteryx *	51	USA: QC. USA: AR, LA, MS, MO, NE, NJ, NC, OH, OK, SC, TN, TX, VA, WI, WV
** Chloroperlidae **		
*Alloperla atlantica* Baumann, 1974	8	USA: NC, WI
*Alloperla caddo* Poulton & Stewart, 1987	2	USA: AR
*Alloperla caudata* Frison, 1934	14	USA: AR, MO, OK
*Alloperla concolor* Ricker, 1935	1	USA: VA
*Alloperla imbecilla* (Say, 1823)	2	USA: PA, WV
*Alloperla neglecta* Frison, 1935	3	USA: TN
*Alloperla ouachita* Stark & Stewart, 1983	1	USA: AR
*Alloperla usa* Ricker, 1952	4	USA: NC, TN, VA, WV
* Alloperla *	3	USA:AR, NC, VA
*Haploperla brevis* (Banks, 1895)	29	Canada: MB. USA: AR, MI, MO, NC, OH, OK, PA, TN, WI, WV
* Haploperla *	2	USA: AR, WI
* Suwallia *	1	USA: CO
*Sweltsa hoffmani* Kondratieff & Kirchner 2009	2	USA: PA, VA
*Sweltsa lateralis* (Banks, 1911)	6	USA: NC, TN, VA
*Sweltsa mediana* (Banks, 1911)	3	USA: VA
*Sweltsa naica* (Provancher, 1876)	1	Canada: PE
*Sweltsa revelstoka* (Jewett, 1955)	1	USA: MT
*Sweltsa urticae* (Ricker, 1952)	2	USA: NC, VA
* Sweltsa *	1	USA: CO
*Triznaka signata* (Banks, 1895)	1	USA: CO
** Peltoperlidae **	43	USA: DE, GA, ID, NC, PA, SC, VA, WA, WV
*Peltoperla arcuata* Needham, 1925	3	USA: TN, WV
*Peltoperla tarteri* Stark & Kondratieff, 1987	1	USA: WV
*Soliperla campanula* (Jewett, 1954)	2	USA: OR
*Tallaperla anna* (Needham & Smith, 1916)	4	USA: NC, VA
*Tallaperla cornelia* (Needham & Smith, 1916)	4	USA: NC, VA
*Tallaperla elisa* Stark, 1983	2	USA: NC
*Tallaperla maria* (Needham & Smith, 1916)	20	USA: GA, NC, TN, VA, WV
* Tallaperla *	2	USA: NJ
*Viehoperla ada* (Needham & Smith, 1916)	8	USA: NC, SC
*Yoraperla brevis* (Banks, 1907)	1	USA: MT
* Yoraperla *	1	USA: CA
** Perlidae **	7	USA: CA, MT, OK, WI
*Acroneuria abnormis* (Newman, 1838)	33	USA: AL,GA, KS, LA, NC, SC, TN, VA, WV, WI
*Acroneuria arenosa* (Pictet, 1841)	31	USA: LA, MS, TX,
*Acroneuria carolinensis* (Banks, 1905)	6	USA: KY, VA, WV
*Acroneuria evoluta* Klapalek, 1909	21	USA: AR, IL, IN, KS, OK, TX*
*Acroneuria filicis* Frison, 1942	6	USA: AR, MO, OH
*Acroneuria frisoni* Stark & Brown, 1991	46	USA: AR, KS, MO, OK, TN, TX*
*Acroneuria internata* (Walker, 1852)	5	USA: MO, NC, VA
*Acroneuria kirchneri* Stark and Kondratieff, 2004	1	USA: WV
*Acroneuria lycorias* (Newman, 1839)	16	CANADA: ON. USA: LA, MI, MN, TX, WI
*Acroneuria perplexa* Frison, 1937	14	USA: AR, OH
* Acroneuria *	32	USA: AR, GA, KS, LA, MO, MT, NH, NC, OK, SC, TN, WV, WI
*Agnetina brevipennis* (Navás, 1912)	1	Mongolia: Bulgan
*Agnetina capitata* (Pictet, 1841)	18	USA: MO, OK, WI
*Agnetina flavescens* (Walsh, 1862)	23	USA: AR, MO, OK, TX*, WI
* Agnetina *	24	USA: LA, MI, OK, WI
*Anacroneuria flavifacies* Jewett, 1958	1	Mexico: Oaxaca
*Anacroneuria litura* (Pictet, 1841)	2	Mexico: Oaxaca, Puebla
*Anacroneuria planicollis* Klapalek 1923	4	Mexico: Puebla, Veracruz
*Anacroneuria quadriloba* Jewett, 1958	3	Mexico: San Luis Potosi
*Anacroneuria wipukupa* Baumann & Olson, 1984	1	USA: AZ
* Anacroneuria *	11	Columbia. Mexico: San Luis Potosi, Veracruz. Panama: Chiriqui. Peru: San Martin. USA: AZ
*Attaneuria ruralis* (Hagen, 1861)	9	USA: AR, FL, KS
*Beloneuria georgiana* (Banks, 1914)	5	USA: NC, SC
*Beloneuria stewarti* Stark & Szczytko, 1976	8	USA: GA, NC, SC, TN
* Beloneuria *	4	USA: NC, SC
*Calineuria californica* (Banks, 1905)	3	USA: MT, OR
*Doroneuria baumanni* Stark & Gaufin, 1974	2	USA: CA, OR
*Eccoptura xanthenes* (Newman, 1838)	17	USA: GA, MS, NC, SC, TN, WV
*Hansonoperla appalachia* Nelson, 1979	1	USA: WV
* Hansonoperla *	1	USA: WV
*Hesperoperla pacifica* (Banks, 1900)	1	USA: MT, WY
*Neoperla carlsoni* Stark & Baumann, 1978	6	USA: AR, LA, TX
*Neoperla catharae* Stark & Baumann, 1978	21	USA: AR, MO, OK, TX
*Neoperla choctaw* Stark & Baumann, 1978	11	USA: AR, OK
*Neoperla clymene* (Newman, 1839)	22	USA: AR, TX
*Neoperla falayah* Stark & Lentz, 1988	2	USA: AR, OK
*Neoperla gaufini* Stark & Baumann, 1978	1	USA: OH
*Neoperla harpi* Ernst & Stewart, 1986	13	USA: AR, MO, OK
*Neoperla occipitalis* (Pictet, 1841)	4	USA: KY, MI*, SC, WI
*Neoperla osage* Stark & Lentz, 1988	1	USA: AR
*Neoperla robisoni* Poulton & Stewart, 1986	13	USA: AR, MO, OK
*Neoperla stewarti* Stark & Baumann, 1978	20	USA: AR, KY, MO, OH, OK
* Neoperla *	166	USA: AR, KS, LA, MO, OK, TX, WI
*Paragnetina fumosa* (Banks, 1902)	31	USA: AL, LA, MS, TX
*Paragnetina immarginata* (Say, 1823)	8	USA: NC, SC, TN, WV
*Paragnetina kansensis* (Banks, 1905)	21	USA: AR, KS, LA, MS, MO
*Paragnetina media* (Walker, 1852)	27	Canada: ON. USA: AR, MI, MN, MO, SC, VA, WI
* Paragnetina *	2	USA: WI
*Perlesta* AR-1 n.sp.	19	USA: AR*--soon to be described
*Perlesta baumanni* Stark, 1989	1	USA: AR
*Perlesta bolukta* Stark, 1989	37	USA: AR, MO, TX
*Perlesta browni* Stark, 1989	8	USA: AR
*Perlesta cinctipes* (Banks, 1905)	12	USA: AR, MO
Perlesta decipiens (Walsh, 1862)	40	USA: AR, MO, OK, TX, WI
*Perlesta ephelida* Grubbs & DeWalt 2012	4	USA: MO
*Perlesta fusca* Poulton & Stewart	1	USA: AR
*Perlesta lagoi* Stark, 1989	9	USA: AR, MO
*Perlesta shubuta* Stark, 1989	1	USA: MO*
* Perlesta *	309	USA: AL, AR, CA, GA, KS, KY, LA, MI, MO, NE, NJ, OK, SC, TX, WI
*Perlinella drymo* (Newman, 1839)	49	USA: AR, KS, LA, MO, OH, OK, TX
*Perlinella ephyre* (Newman, 1839)	24	USA: AR, KS, LA, MO, OK, SC
* Perlinella *	2	USA: IL
** Perlodidae **	11	USA: GA, NC, OK, SC
*Arcynopteryx dichroa* (McLachlan, 1872)	2	USA: AK, MI
*Calliperla luctuosa* (Banks, 1906)	3	USA: OR
*Cascadoperla trictura* (Hoppe, 1938)	2	USA: OR
*Chernokrilus misnomus* (Claassen, 1925)	1	USA: OR
*Clioperla clio* (Newman, 1839)	146	USA: AR, AL, CT, DE, IL, IN, KY, MS, MO, NC, OH, OK, PA, SC, TN, VA, WI, WV
*Cultus pilatus* (Frison, 1942)	6	Canada: BC. USA: OR.
*Cultus verticalis* (Banks, 1920)	1	USA: NC
* Cultus *	5	USA: GA, NC, TN
*Diploperla duplicata* (Banks, 1920)	4	USA: MS, PA, SC, TN
*Diploperla morgani* Kondratieff & Voshell, 1979	3	USA: VA
*Diploperla robusta* Stark & Gaufin, 1974	11	USA: OH, WV
* Diploperla *	4	USA: TN, WV
*Diura bicaudata* (Linnaeus, 1758)	3	Canada: YT. United Kingdom: England
*Diura knowltoni* (Frison, 1937)	3	USA: CO, OR
*Diura washingtoniana* (Hanson, 1940)	1	USA: NH
*Helopicus bogaloosa* Stark & Ray, 1983	2	USA: MS
*Helopicus nalatus* (Frison, 1942)	13	USA: AR, KS, MI, MO, OK
*Helopicus subvarians* (Banks, 1920)	9	USA: LA, SC, TN, VA
*Hydroperla crosbyi* (Needham & Claassen, 1925)	114	USA: AR, IN, KS, OK, TX
*Hydroperla fugitans* (Needham & Claassen, 1925)	19	USA: IL, KS, MO, TN, TX
*Isogenoides doratus* (Frison, 1942)	2	USA: IA, MI
*Isogenoides elongatus* (Hagen, 1874)	3	USA: ID, MT
*Isogenoides hansoni* (Ricker, 1952)	5	USA; NY, PA, WV
*Isogenoides olivaceus* (Walker, 1852)	13	USA: MI, WI
*Isogenoides varians* (Walsh, 1862)	3	USA: IL, MS, VA
*Isogenoides zionensis* Hanson, 1949	1	USA: NM
* Isogenoides *	3	Canada: NS. USA: MI
*Isoperla bifurcata* Szczytko & Stewart, 1979	1	USA: CA
*Isoperla bilineata* (Say, 1823)	62	USA: IL, IN, IA, KS, LA, MI, MN, MS, MO, NE, ND, OH, WI
*Isoperla burksi* Frison, 1942	15	USA: AR, IL, OH, VA
*Isoperla cotta* Ricker, 1952	22	USA: MI, WI
*Isoperla davisi* James, 1974	85	USA: AL, AR, DE, GA, LA, MS, TX
*Isoperla decepta* Frison, 1935	38	USA: IL, IN, MI, MO, OH, OK, VA
*Isoperla dicala* Frison, 1942	90	Canada: ON. USA: AL, CT, GA, ME, MI, MN, MO, NC, OH, PA, SC, TN, VA, WV, WI,
*Isoperla distincta* Nelson, 1976	1	USA: TN
*Isoperla francesca* Harper, 1971	1	USA: VT*
*Isoperla frisoni* Illies, 1966	38	Canada: ON, QC. USA: IN, MA, MI, MN, NY, SC, TN, WI
*Isoperla fulva* Claassen, 1937	2	USA: WA
*Isoperla grammatica* (Poda, 1761)	2	France, United Kingdom
*Isoperla gravitans* (Needham & Claassen, 1925)	1	USA: OR
*Isoperla holochlora* Klapalek, 1923	40	USA: AL, DE, ME, NC, PA, SC, TN, VA, WV
*Isoperla irregularis* (Klapalek, 1923)	133	USA: AR, IL, KS, LA, MO, OK, TX,
*Isoperla jamesae* Grubbs & Szczytko, 2010	1	USA: AL
*Isoperla lata* Frison, 1942	13	Canada: QC. USA: MI, WI
*Isoperla longiseta* Banks, 1906	2	USA: TX
*Isoperla marlynia* (Needham & Claassen, 1925)	44	Canada: MB. USA: IL, IN, KS, MI, NE, NJ, PA, SC, VA, WI
*Isoperla montana* (Banks, 1898)	15	USA: ME, MA*, NY, NC, PA, OK*, SC, VA, VT*, WV
*Isoperla mormona* Banks, 1920	1	USA: WA
*Isoperla namata* Frison, 1942	104	USA: AR, IN, MO, OK,
*Isoperla nana* (Walsh, 1862)	97	USA: IL, IN, MI, NY, OH, PA, WI
*Isoperla obscura* (Zetterstedt, 1840)	1	France
*Isoperla orata* Frison, 1942	14	USA: NH, NY, NC, TN, VT
*Isoperla ouachita* Stark & Stewart, 1973	73	USA: AR, MO, OK
*Isoperla petersoni* Needham & Christenson, 1927	2	USA: UT, WY
*Isoperla pseudosimilis* Szczytko & Kondratieff, 2015	1	USA: TN
*Isoperla quinquepunctata* (Banks, 1902)	8	USA: MT, NE, NM, SD
*Isoperla richardsoni* Frison, 1935	25	USA: AR, C, IL, MN, MO, WI
*Isoperla sagittata* Szczytko & Stewart, 1976	1	USA: TX
*Isoperla signata* (Banks, 1902)	120	USA: AR, CT, MI, MN, MO, NH*, NY, OK, PA, VA, WI
*Isoperla similis* (Hagen, 1861)	5	USA: CT, MD, PA, VT*, VA
*Isoperla slossonae* (Banks, 1911)	50	Canada: NS. USA: ME, MI, MN, NH, WI
*Isoperla sobria* (Hagen, 1874)	4	Canada: AB, BC. USA: UT, WY
*Isoperla szczytkoi* Poulton & Stewart, 1987	7	USA: AR
*Isoperla transmarina* (Newman, 1838)	50	Canada: MB, ON, SK. USA: ME, MI, WI
*Isoperla viridinervis* (Pictet, 1865)	1	France
*Isoperla zuelligi* Szczytko & Kondratieff, 2015	2	USA: AL, NH*
* Isoperla *	131	Canada: ON, QC. USA: AL, AK, CA, FL, GA, IL, IN, KS, LA, MA, MN, MO, NE, NH, NC, OH, OK, PA, SC, TN, TX, VA, WI, WV
*Kogotus modestus* (Banks, 1908)	5	USA: CO, MT, WY
*Kogotus nonus* (Needham & Claassen, 1925)	8	USA: OR
*Malirekus hastatus* (Banks, 1920)	16	USA: KY, NC, TN, VA, WV
* Malirekus *	7	USA: NY, NC, PA, VA
* Megarcys *	4	USA: CO, OR, WA
*Oconoperla innubila* (Needham & Claassen, 1925)	4	USA: NC
*Oroperla barbara* Needham, 1933	2	USA: CA
*Osobenus yakimae* (Hoppe, 1938)	2	USA; CA, WA
*Perlinodes aureus* (Smith, 1917)	3	USA: OR, WA
*Pictetiella expansa* (Banks, 1920)	2	USA: CO
*Remenus bilobatus* (Needham & Claassen, 1925)	10	USA: MD, PA, TN, WV
* Remenus *	2	USA: SC, TN
*Setvena bradleyi* (Smith, 1917)	2	Canada: BC. USA: MT
*Setvena tibialis* (Banks, 1914)	2	USA: MT
*Skwala americana* (Klapalek, 1912)	4	USA: OR, UT
*Yugus arinus* (Frison, 1942)	5	USA: NC, TN, VA
*Yugus bulbosus* (Frison, 1942)	2	USA: TN
*Yugus kirchneri* Nelson, 2001	10	USA: VA, WV
* Yugus *	5	USA: NC, WV
** Pteronarcyidae **		
*Pteronarcella badia* (Hagen, 1874)	1	USA: UT
*Pteronarcys biloba* Newman, 1838	4	USA: WV
*Pteronarcys californica* Newport, 1851	1	Canada: BC
*Pteronarcys dorsata* (Say, 1823)	26	Canada: BC, ON. USA: LA, MS, WI
*Pteronarcys pictetii* Hagen, 1873	29	USA: AR, IL, MO, WI
*Pteronarcys proteus* Newman, 1838	1	USA: WV
*Pteronarcys scotti* Ricker, 1952	4	USA: TN, VA
* Pteronarcys *	25	USA: GA, KS, NC, NJ, SC, TN, TX, VA, WI, WV
** TRICHOPTERA **		
** Hydropsychidae **		
* Arctopsyche *	1	USA: OR
*Parapsyche cardis* HH Ross, 1938	1	USA: VA
** Philopotamidae **		
*Dolophilodes distincta* (Walker, 1852)	1	USA: VA
** Rhyacophilidae **		
* Rhyacophila *	1	USA: OR

## Hosting institution

University of Illinois Urbana-Champaign, Prairie Research Institute, Illinois Natural History Survey, Champaign, Illinois, USA 61820.
